# Assessing Collaboration in a National Research Partnership in Quality Improvement in Indigenous Primary Health Care: A Network Approach

**DOI:** 10.3389/fpubh.2018.00182

**Published:** 2018-06-25

**Authors:** Frances C. Cunningham, Veronica Matthews, Anna Sheahan, Jodie Bailie, Ross S. Bailie

**Affiliations:** ^1^Wellbeing and Preventable Chronic Diseases Division, Menzies School of Health Research, Charles Darwin University, Spring Hill, QLD, Australia; ^2^University of Sydney, University Centre for Rural Health, Lismore, NSW, Australia; ^3^Queensland Aboriginal and Islander Health Council, South Brisbane, QLD, Australia

**Keywords:** indigenous health, primary health care, quality improvement, partnerships, network analysis

## Abstract

**Background:** The ABCD National Research Partnership was formed in mid-2010 as a collaboration to harness the expertise, experiences and resources of Aboriginal and Torres Strait Islander community-controlled peak bodies, government and research organisations to improve the quality of Indigenous primary health care. The aim of this study was to apply social network methods to assess collaboration and functioning of the Partnership at two time-points.

**Methods:** A social network analysis (SNA) survey was conducted in early 2013, with a follow-up survey in mid-2014. In the two survey rounds, online surveys were emailed to one senior person of the organisation participating in the Partnership (2013: 14 organisations; 2014: 11 organisations). The surveys collected data on respondent perceptions of the Partnership as well as social network relationship data. Social network methods were used to apply standardised metrics to assess how well the partnership was functioning as a collaborative three years into its operation, and in its fourth year.

**Results:** Most respondents rated the Partnership as successful in progressing toward its goals. Network density and centrality scores show a well-connected partnership spanning different organisational types and states/territories (Northern Territory, Queensland, Western Australia, South Australia, and Far-West New South Wales). High centrality scores reflect high connectivity between key hubs in the network, contributing toward the shared goal of improved Indigenous primary health care. Network diagrams show key structural positions by organisational type, the frequency and intensity of interactions and the strengths and potential vulnerabilities in the partnership network, with comparisons at two time points for the partnership.

**Conclusions:** The study found that the Partnership was effective in securing collaboration across its partners. Partners' contribution of resources reflected their active involvement. There was a high level of agreement on the achievement of the key goals of the Partnership, showing shared sense-making amongst partners. SNA tools assisted with monitoring the network over time to develop strategies supporting connections between partners for sustaining collaborative learning. Study findings identify successful approaches for a research partnership to improve quality of care in Indigenous primary health care and provide encouragement for wider applications for research partnerships and collaborations in Australia and internationally.

## Introduction

The role of collaborative and partnership research approaches has been increasingly acknowledged and accepted across all sectors, including the health sector, with applications extending to partnerships to improve patient outcomes through applied health research. Such collaborative research is designed to bring together those who study societal problems (researchers) with those who act on those problems (decision-makers, practitioners, and patients) (1), as a means of increasing the relevance and use of health research (2, 3) and of fostering knowledge transfer and implementation (4–6). The aim of this study was to apply network methods to evaluate collaborative aspects of a research partnership at two time-points, mid-term and in its final year of operation.

A research partnership is defined in this paper as having a shared governance and management structure between the partners to achieve shared goals. Much is known about partnership characteristics and factors that are needed for successful relationships (6). Genuine respect, trust and goodwill between all parties, built over time, are essential for constructive relations and a collaborative partnership (5, 7, 8); sustained contact is required, preferably including face-to-face meetings (9) to facilitate information sharing (10, p. 94); and recognition not only of the mutual benefits of the partnership, but a shared belief in higher level outcomes and impacts beyond the partnership itself (11). A common language (12), a shared vision and goals can increase a sense of commitment among members (8, 13, 14). Partnerships should have the capacity and capabilities to engage, an equitable learning culture, and strong leadership that is inclusive (11, 15). It is important that there are clear roles and expectations for all partners that accommodate different needs and capabilities (5) and a positive culture, and organisational structures that support expectations for change and new behaviours (4–6, 16).

There are lessons for partnerships from other associated network structures, including health networks and collaboratives. While cohesive and collaborative health professional networks can facilitate the coordination of care, potential barriers to their effectiveness and sustainability include cliques and over-reliance on central agencies, sponsors or individuals (17). Sponsors and support organisations can form a “glue” to hold the partnership together (18, 19), however partnership sustainability may be reliant on continuity of such support. The term, quality improvement collaborative, is used for different multifaceted packages that focus on accelerating better outcomes, with a well-known example being that developed by the US Institute of Healthcare Improvement (20). Implementation of collaboratives is complex and they may not achieve their desired outcomes (21). Key quality improvement collaborative success factors relate to who should be included, what the focus should be, how the collaborative should be run, and what resources are needed (9). To succeed, they must be well planned and resourced, encompass passionate professionals and leaders, have realistic expectations and be given enough time to show an impact (9). In addition to success factors relating to characteristics of partnerships and collaboratives themselves, wider factors may be at work in the health system in which they operate. Local and national policies and drivers, incentive schemes, staffing levels and priorities and many other external factors are likely to have an impact (9).

Although collaborative and partnership research approaches have become widely accepted for the benefits that accrue from such collaboration, little research has examined the mechanisms through which partnerships function (21, 22). It is therefore important to evaluate the functioning of research partnerships. Such evaluations may be conducted at different times in the partnership's life-cycle. Assessment in the early stages assists with determining whether the partnership is working well, and whether aspects of its resources and processes need to be addressed. Thence, strategies may be developed to facilitate achievement of partnership goals. Toward the end of the lifecycle, the focus of the evaluation will be on how well the partnership has worked in achieving its objectives.

The use of social network analysis (SNA) can help in identifying the underlying network of relationships in a collaboration that people rely on to find information and to solve problems. In their classic work, Cross et al. (23) show the benefit of using SNA to identify the often informal, or invisible, networks in organisations, thereby enabling employees to effectively collaborate and integrate disparate expertise. SNA can be an invaluable tool for visualising the structure of a collaboration, and in identifying key roles, information flows and collaboration, and potential structural weaknesses in the network.

Aboriginal and Torres Strait Islander people (hereafter respectfully referred to as Indigenous) remain the least healthy subpopulation in Australia, with a life expectancy at birth of around 10 years less than non-Indigenous Australians (24). Primary health care (PHC) is recognised as central not just to dealing directly with chronic disease but also for providing a multidisciplinary framework to interface with other sectoral domains and tackle Indigenous disadvantage (25, 26). While PHC has improved health outcomes of populations in general, there is evidence that better access to PHC has also improved health outcomes for Indigenous populations (27, 28).

To address the need to improve the quality of care in Indigenous PHC settings, the Audit and Best Practice for Chronic Disease (ABCD) Project was established by Menzies School of Health Research and partners in the Northern Territory in 2002, and subsequently extended. From 2010 to 2014, the ABCD National Research Partnership (“the Partnership”) was established to build on collaborative relationships of the ABCD Project across the national network of Indigenous health care centres committed to continuous quality improvement (CQI) (29). Bailie et al. describe the Partnership as a model for large-scale change to achieve population health outcomes and provide further detail on the ABCD program of work (30). Under the Partnership, 175 PHC centres—including Aboriginal Community Controlled Health Organisations (ACCHOs) and government-managed health centres—provided the Partnership with de-identified clinical audit data derived from the use of CQI tools and processes for its research database.

Partners from each participating jurisdiction included: ACCHO peak bodies, Government health departments, and a lead research institution. The Partnership was managed by a Project Coordinating Centre, based at Menzies School of Health Research, governed by the Project Management Committee. Initial partners included: for the Northern Territory, the Aboriginal Medical Services Alliance Northern Territory, the Northern Territory Department of Health and the Menzies School of Health Research; for Queensland, the Queensland Aboriginal and Torres Strait Islander Health Council, Queensland Health, James Cook University and the University of Queensland; for Western Australia, Curtin University and the Combined Universities Centre for Rural Health; for South Australia, the Aboriginal Health Council of South Australia, the South Australian Department of Health and the University of South Australia; and for New South Wales, Maari Ma Health Aboriginal Corporation. Over its duration three partners did not continue their active engagement in the Partnership (University of Queensland, Curtin University, University of South Australia).

The Partnership applied participatory action research approaches to: (1) investigate variation in quality of care between PHC centres and between regions; (2) explore factors associated with clinical performance of PHC centres at the health centre and regional level; (3) identify and examine specific strategies that have been effective in improving PHC clinical performance; and (4) work with health service staff, management and policy makers to enhance the effective implementation of successful strategies.

This study applies SNA to evaluate collaborative aspects of the Partnership at two timepoints. It contributes to a wider assessment of the Partnership (31), and to the literature on applied health research partnerships and collaborations, adding to our understanding of enabling factors for successful health research partnerships.

## Methods

### Study design

Traditional survey methods were combined with SNA tools to assess Partnership functioning through two cross-sectional surveys, conducted in early 2013 after 3 years of operation, and in mid-2014, the fifth and final year. The 2013 survey was designed: (1) to assess whether the structure and processes of the Partnership were facilitating progress toward project goals; (2) to identify any issues for the Partnership to address; and (3) to determine what strategies should be developed to address any deficiencies. The final year survey provided: (1) a follow-up assessment of the Partnership in its last year of operation using the same survey questions and (2) feedback on the overall evaluation of the Partnership through additional items. These items obtained partner feedback on Partnership performance in relation to its goals. Survey items (for 2013 and 2014) are provided in Table 1 in Appendix A of Supplementary Material.

The survey was adapted from the open-access web resource “Partner” tool (32). “Partner” is a SNA-based tool, specifically developed by Varda and colleagues for use by public health agencies in the United States to assess and evaluate collaboration between public health organisations (22, 33). This tool allows for the network survey to be sent to a representative of each partner organisation. The survey approach was presented and discussed with partners at the biannual Partnership meeting in Darwin, December 2012, and the survey was amended to incorporate partner feedback and advice.

### Ethics

This research was conducted under ethics approvals granted to the ABCD Partnership Project (Human Research Ethics Committee of the Northern Territory Department of Health and Families and Menzies School of Health Research (EC00153), Central Australian Human Research Ethics Committee (12–53), Queensland Health (HREC/11/QTDD/47), South Australian Aboriginal Health Research Ethics Committee (04-10-319), the Greater Western Area Health Service, Human Research Ethics Committee, New South Wales Health (11/GWAHS/23), and the Western Australian Aboriginal Health Ethics Committee (WAAHEC 111-8/05).

### Participants and survey administration

For a number of reasons, we restricted the network analysis to the 14 formal signatories in the Partnership, using a “bounded network” SNA sampling approach. These reasons included ethical approval and resource constraints. However, over the Partnership's lifecycle there was engagement with many more collaborators who were involved, for example, in analyzing data in the Partnership database for specific clinical care areas, or collaborating with Partnership researchers. Although the Partnership network included a relatively small number of organisations, a number of these are very large organisations with large workforces (e.g., one organization had over 60,000 employees at the time of the study). The partners were also geographically diverse, representing a majority of the Australian states and territories.

In both survey rounds, an email invitation to participate in this survey was sent to the most senior person with direct involvement in the Partnership from each partner organisation. The first survey was distributed in January 2013. Respondents completed the survey online over a 2-week period, with weekly emailed reminders, and telephone follow-up where required. The second survey was distributed in May 2014, also with emailed reminders and telephone follow-up where required.

### Analysis

Partnership network data from the two surveys were collected using the “Partner” tool and were analysed through an electronic database using SNA methods. Network graphs were generated as well as a range of network metrics. SNA tools permit visualisation of the organisations in the network and their connections through network maps. Several network measures (defined in Table 1) were used to assess the Partnership from a network perspective.

**Table 1:** Definition of network measures.

**Measure**	**Definition**
Network density	General level of linkage, or cohesion, among network members; reflects total number of links, or “ties,” between organisations as a proportion of the maximum number of ties within the entire network.
Degree centralisation	How tightly the organisations in the Partnership are connected around the most central point of the network and how reliant the network may be on a central hub.
Frequency of interactions	Types and levels of communication among partners.
Depth of interactions	“Networking” is the basic form of interaction, with little communication and no shared decision-making. “Collaborative activity,” the next level involves regular communication, and sharing information and resources with some shared decision-making.
Value	A measure of perceived power, level of involvement and resource contribution.
Trust	A measure of reliability, shared belief in mission, and opportunity for frank discussion.

In 2013, a written survey report was provided to partner organisations who gave feedback on the results to the Partnership to assist it in addressing strategies for communicating with and providing information to partners. For example, the Partnership developed processes to disseminate its research findings through reports, evidence briefs and summary impact reports. A further report was provided to partner organisations on the comparative results of the two surveys in 2014. Findings were presented, and there was a process of discussion, feedback and reflection with partners at the Brisbane October 2014 biannual meeting.

## Results

### Survey participation

For the 2013 survey, 11 out of 14 Partnership organisations responded, including four ACCHOs, three state/territory government health departments and four research organisations. Two research organisations did not participate in the survey and another did not fully complete the survey. Therefore, figure descriptors provide the respondent numbers for the specific survey items. Non-respondents' linkage nodes are derived from responses of other respondents and are included in the figures to reflect the partner network. At the time of the 2014 survey, two partners were no longer active participants in the Partnership and 11 out of the 12 active Partnership organisations responded to the survey. This included four ACCHOs, three state/territory government health departments and four research organisations.

### Partners' overall perceptions of partnership

Table 2 shows partner representatives' perceptions of the Partnership regarding goals and level of success. The most important outcome or goal was identified by most respondents as “*providing a better understanding of how CQI process and tools can be designed and supported to suit local service needs*,” and then “*improved PHC services for Indigenous communities*,” followed by “*increased knowledge sharing across the Indigenous PHC sector.”* Most partners selected outcomes relevant to the broader benefit of Indigenous communities.

**Table 2:** Perceptions of Partnership, 2013 and 2014.

**Item**	**Most highly rated selections**	**2013 (*n* = 12)**	**2014 (*n* = 11)**
Goals of partnership	Providing a better understanding of how CQI processes and tools can be designed and supported to suit local service needs	12	9
	Improved PHC services for Indigenous communities	8	9
Most important outcome or goal	Providing a better understanding of how CQI processes and tools can be designed and supported to suit local service needs	3	5
	Improved health outcomes for Indigenous communities	5	2
	Increased knowledge sharing across the Indigenous PHC sector	2	1
Overall level of success of Partnership	Successful or very successful	7	8
Main attribute contributing to Partnership success	Exchanging information/knowledge	9	8
	Partners having the opportunity to guide research and development efforts	7	9
	Informal relationships created	5	8
Resource contributions from each Partner	Leadership/advocacy	10	8
	Specific health expertise	10	8
	In-kind resources	10	6
	Staffing resources	6	8
Partner's most important resource contribution	Staffing resources	2	4
	Specific health expertise	2	3
	Data resources, including data sets, collection and analysis	2	2
	Leadership/advocacy	3	1

Most respondents rated the Partnership as “successful” or “very successful” in progressing toward its goals. None of the partners rated the Partnership as “not successful” in either year. When asked about the main Partnership attribute contributing to its success, items most highly ranked were “*exchanging information/knowledge,”* “*partners having the opportunity to guide research and development efforts,”* and “*informal relationships created.”*

Respondents ranked four items as their most important resource contributions: “*staffing resources,” “specific health expertise,” “data resources, including data sets, collection and analysis,” and “leadership/advocacy.”* The range of contributions selected by partners shows that contributions can be broader and more balanced by having a range of stakeholders involved in a collaboration, thereby also enhancing capacity to achieve overall objectives.

As shown in Table 3, in the 2014 survey 10 respondent partners provided feedback on their assessment of the usefulness to their work of key processes used throughout the Partnership, and assessed the Partnership outputs. The biannual Partnership meetings were ranked most highly as very or fairly beneficial by respondents, followed by Partnership reports and publications, and thirdly State and Territory steering committee meetings. All respondents perceived that “*informal contact with colleagues through the Partnership”* was either fairly or very beneficial. Ranked secondly, both “*meeting new colleagues through the Partnership”* and “*formal communication with colleagues through the partnership”* were either fairly or very beneficial.

**Table 3:** Perceptions of Partnership Achievements: 2014 (*n* = 10).

**Item**	**Most highly rated selections**	**Rated fairly or very beneficial**
Perceptions of Partnership processes and outputs	Biannual Partnership Meetings	9
	Partnership Reports and Publications	7
	State/Territory Steering Committee Meetings	6
Perceived benefits of collaboration	Informal contact with colleagues through Partnership	10
	Meeting new colleagues through Partnership	9
	Formal communication with colleagues through Partnership	9
Partners' perceptions of achievement of primary goals	Investigation of variation in quality of care between PHC centres and between regions	9
	Exploration of the factors associated with clinical performance of PHC centres at health centre and regional levels	9
	Identification and examination of specific strategies that have been effective in improving PHC clinical performance	7
Partner's perceptions of achievement of secondary goals	Capacity building for those working on quality improvement in Indigenous PHC	6
	Fostering efficient and effective exchange and use of data/information among service providers and policy makers for integrated quality improvement and improved Indigenous health outcomes	5

The goal of “*investigation of the variation in quality of care between PHC centres and between regions”* was ranked most highly by respondents as being fairly or very successful. Similarly, the goal of “*exploration of the factors associated with clinical performance of PHC centres at the health centre and regional level”* was also equally ranked by respondents as being fairly or very successful. For its secondary goals, most respondents perceived the Partnership fairly or very successful in “*capacity building for those working on quality improvement in Indigenous health care.”*

### Examination of network structure and features

#### Network structure

**Figure 1:**
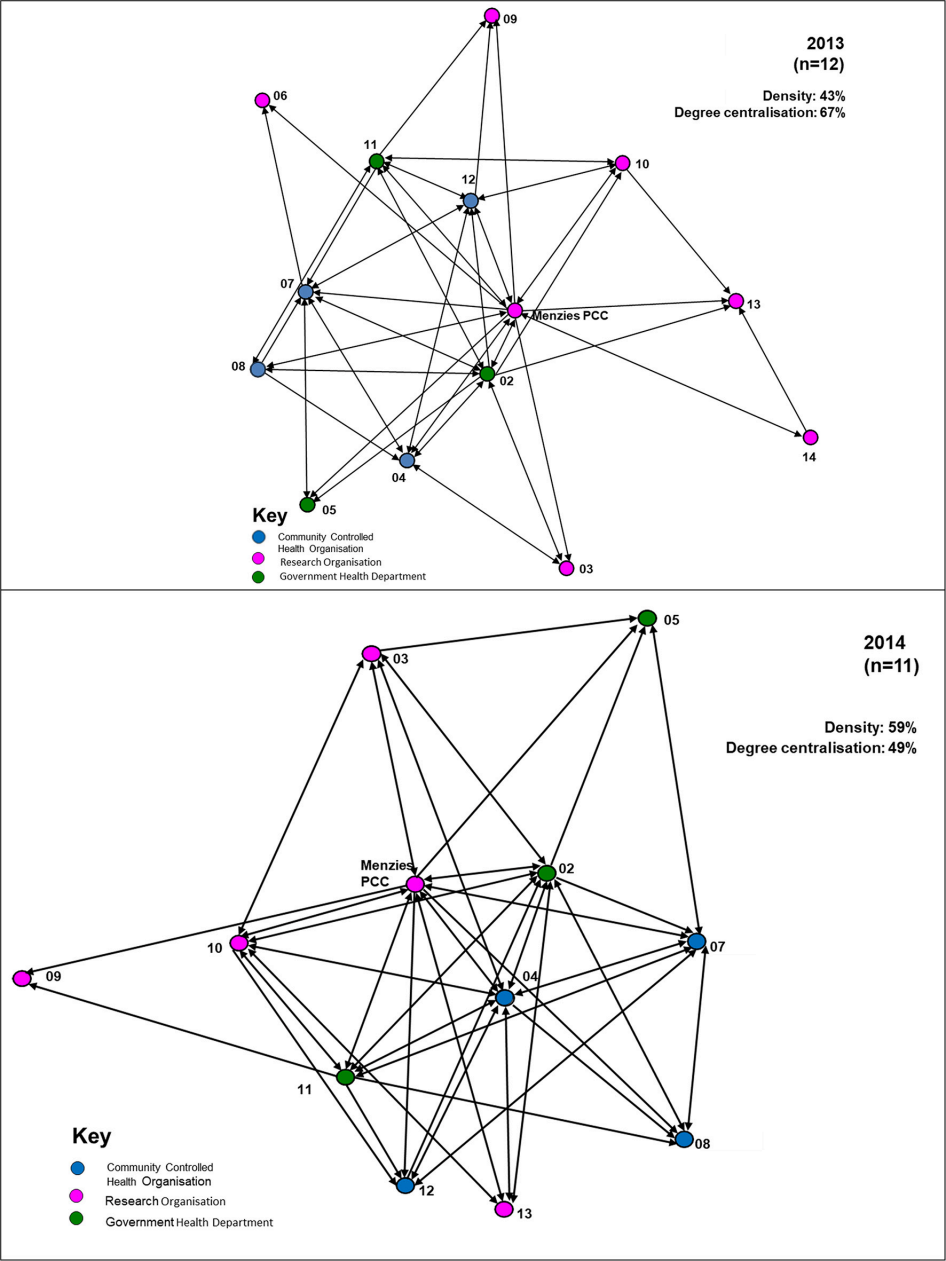
Network diagrams of the Partnership showing ties between organisations, 2013, 2014.

Analysis of network data shows an increase in the network *density* score from 0.43 in 2013 to 0.59 in 2014 (Figure 1). In 2013, the Partnership was relatively well connected with ~43% of all possible links existing between partner organisations, including connections across jurisdictional boundaries. For 2014, Figure 1 shows linkages of the 12-partner network, compared with 14 partners in 2013. In 2014, with 59% of all possible links existing between partners, there was a higher level of connectivity compared with 2013. In the network diagrams, apart from the Project Coordination Centre (PCC) partner nodes are represented by deidentified codes, and the nodes are colour-coded by type of organisation.

The *degree centralisation* score of the Partnership network was 0.67 in 2013, compared with 0.49 in 2014, reflecting a decrease in the level of centralisation. Within both the 2013 and 2014 networks there are some central positions (nodes), with a higher proportion of connections compared to other organisations. For example, in 2013, the Project Coordination Centre connected to all other members (13/13 connections), and similarly in 2014 (11/11 connections). This reflects the important role of the Centre as a central network coordinator, key player or “broker.” In 2013 there were four other agencies with at least seven or more connections. There were five other agencies with at least seven or more connections in 2014, reflecting an increase in the role of other agencies as significant communication connectors in the network.

In Figure 1, the arrowed links reflect the direction of the connections in the network, showing whether the ties are bi-directional, with reciprocity, or uni-directional and asymmetric. Such information was useful for feedback to network members, and for consideration by the network itself, as this provided a picture of communication flows and was an indicator of where there might be a need for strategies to ensure better linkages with network members, where improvement was seen as being important. This information was useful, for example, for those involved in coordinating the regional hubs of the Partnership.

#### Network communication

**Figure 2:**
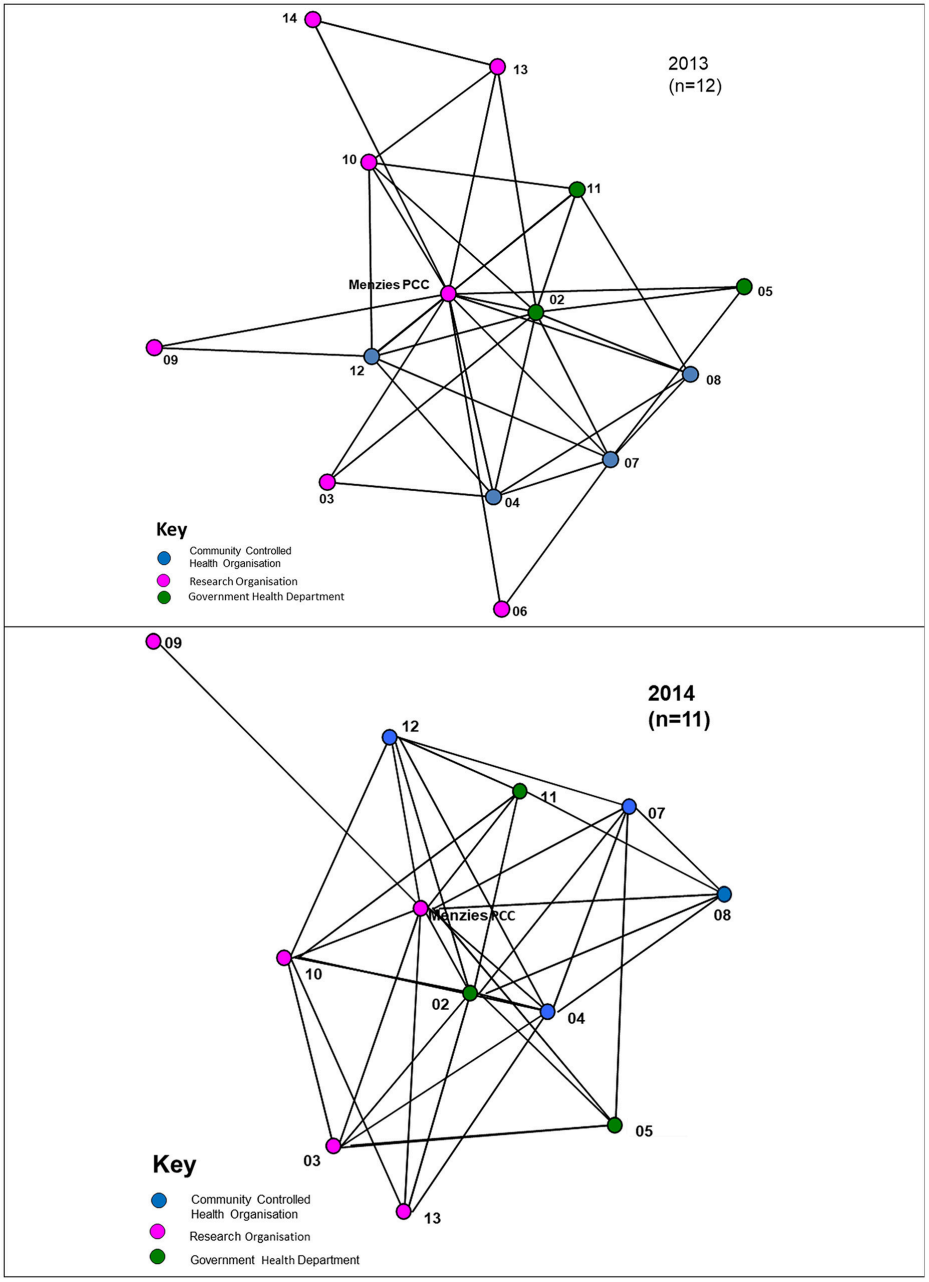
Links between organisations that communicated at least twice per year, 2013, 2014.

**Figure 3:**
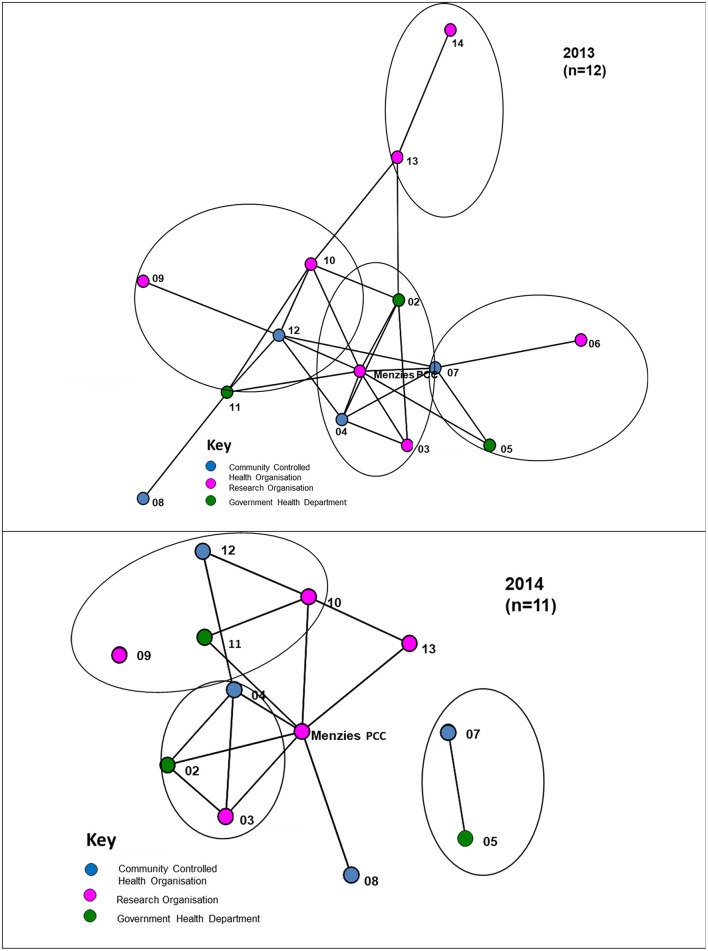
Links between organisations that communicated at least monthly (circles represent different jurisdictions), 2013, 2014.

Most interactions between organisations occurred at least twice a year, reflecting regular bi-annual Partnership face-to-face meetings (Figure 2). For interactions that occurred at least monthly, a number of the cross-jurisdictional links disappear with a majority of the connections occurring within regional hubs of the Partnership, as circled in Figure 3. While four regional hubs were identified as active in 2013, only three regions identified as active in 2014, as a partner in the fourth region was no longer involved in 2014. The circles around these jurisdictions indicate connections which occurred most frequently within the regions on a monthly basis, whereas connectivity across regions is seen less frequently (twice a year). This may have been due to the regional steering committees of the Partnership which met on a regular basis, up to once a month.

Figure 3 shows that in 2014 there was less frequent communication between partners on at least a monthly basis, with isolates appearing (that is, those organisations that have no reported connections in the project). Whereas twice-yearly meetings of the partnership were held in previous years, in 2014 only one face-to-face meeting of partners was held because of resource constraints This is likely to have impacted on the difference in communication results in the two surveys.

#### Depth of interaction

**Figure 4:**
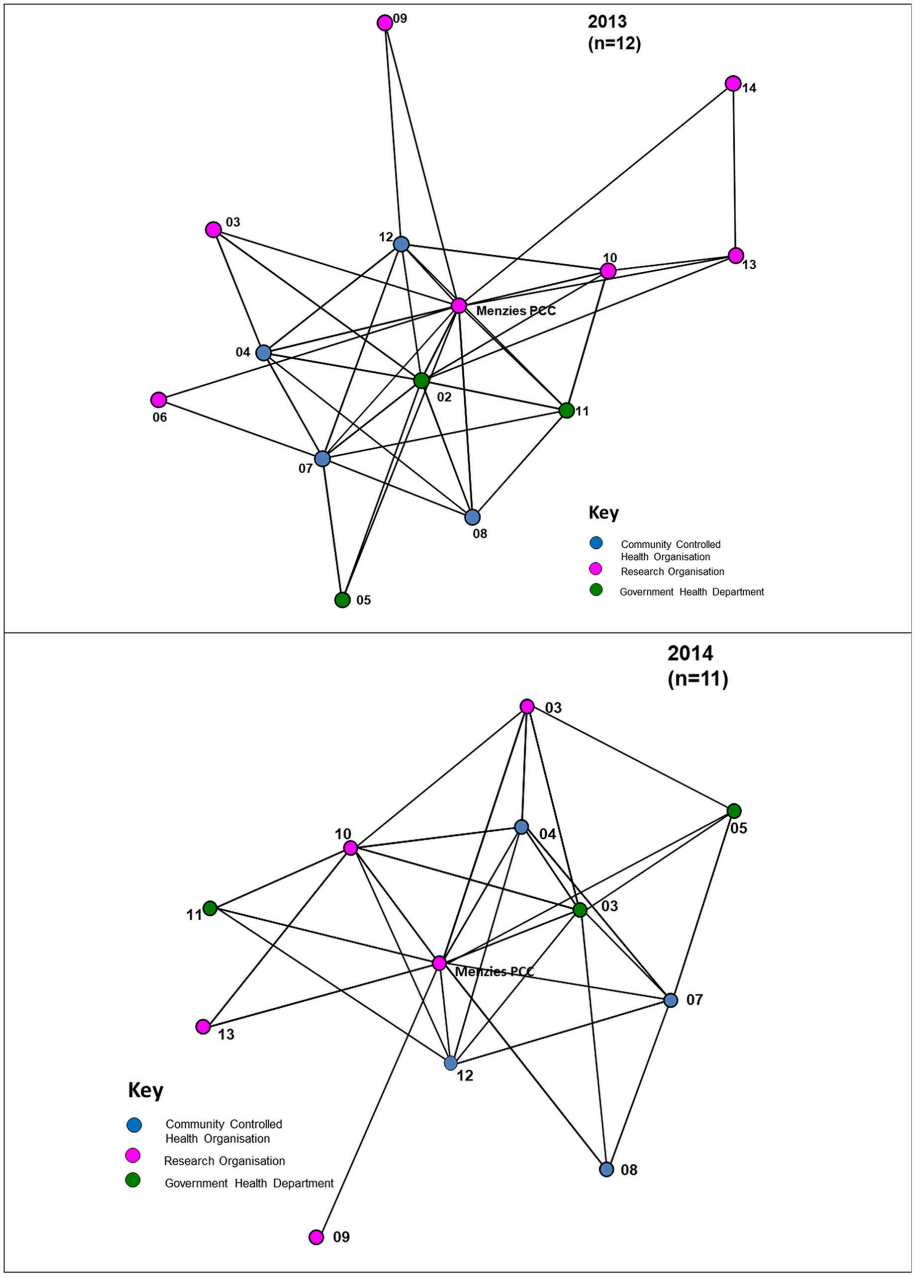
“Networking” interactions with other organisations in the Partnership, 2013, 2014.

**Figure 5:**
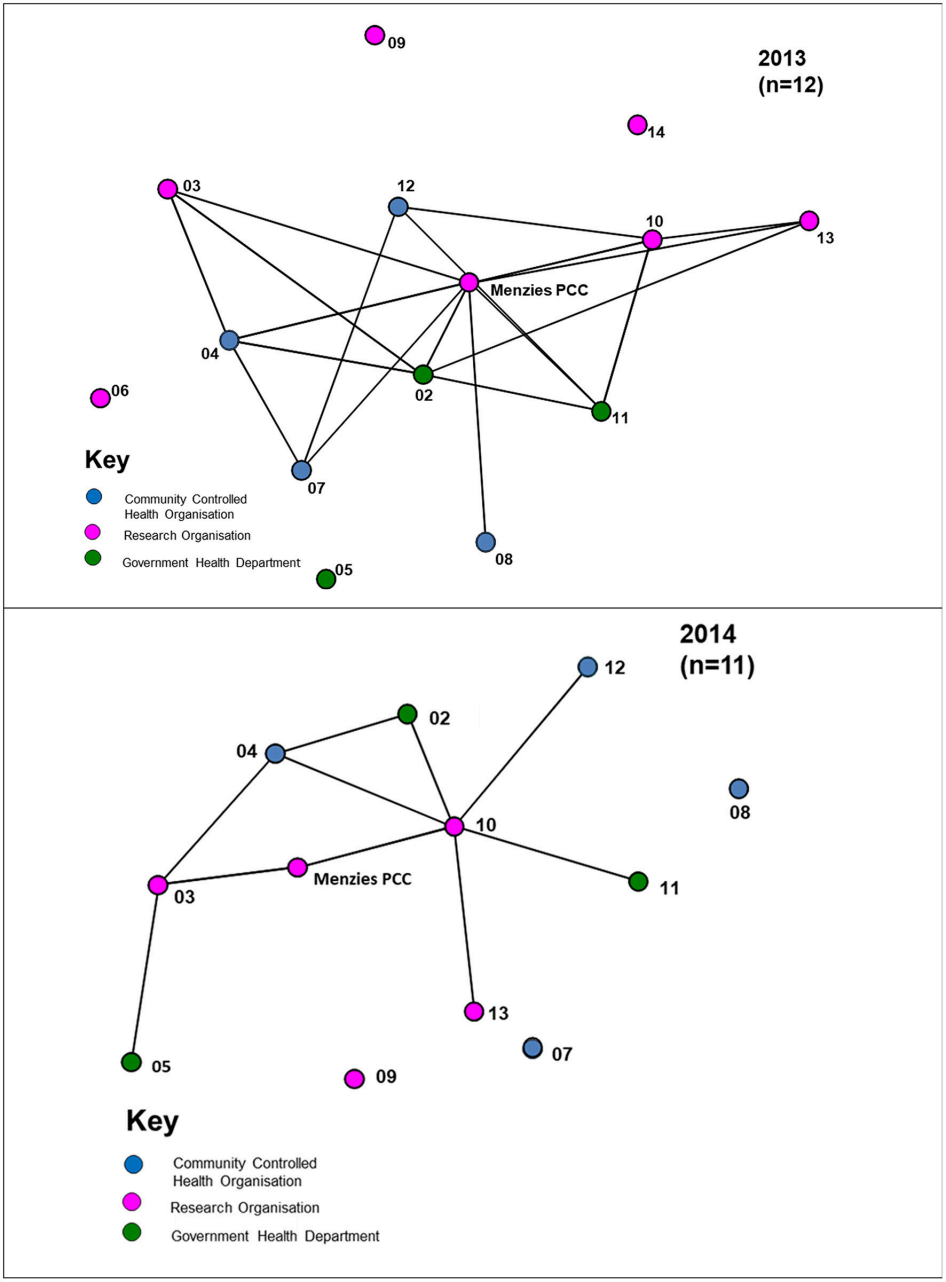
“Collaborative” interactions with other organisations in the Partnership, 2013, 2014.

The different depths of interaction between organisations for networking and coordinated activities, respectively, are shown in Figures 4, 5. The first, “*networking”* level, shows the most basic form of interaction involving an awareness of the organisation, little communication and no shared decision-making. The next level of interaction, “c*ollaborative activity,”* involves regular communication, and sharing information and resources with some shared decision-making to enhance capacity for the mutual benefit of the Partnership project. At this level “*isolates”* (nodes with no connections) appear. As shown in Figure 5, at least one isolate occurs in three of the jurisdictions in 2013. Two of these isolates were non-respondents to the 2013 survey. In 2014, there is similarly at least one isolate in three of the jurisdictions, with one of these being a non-respondent to the survey.

#### Value and trust of members

**Figure 6:**
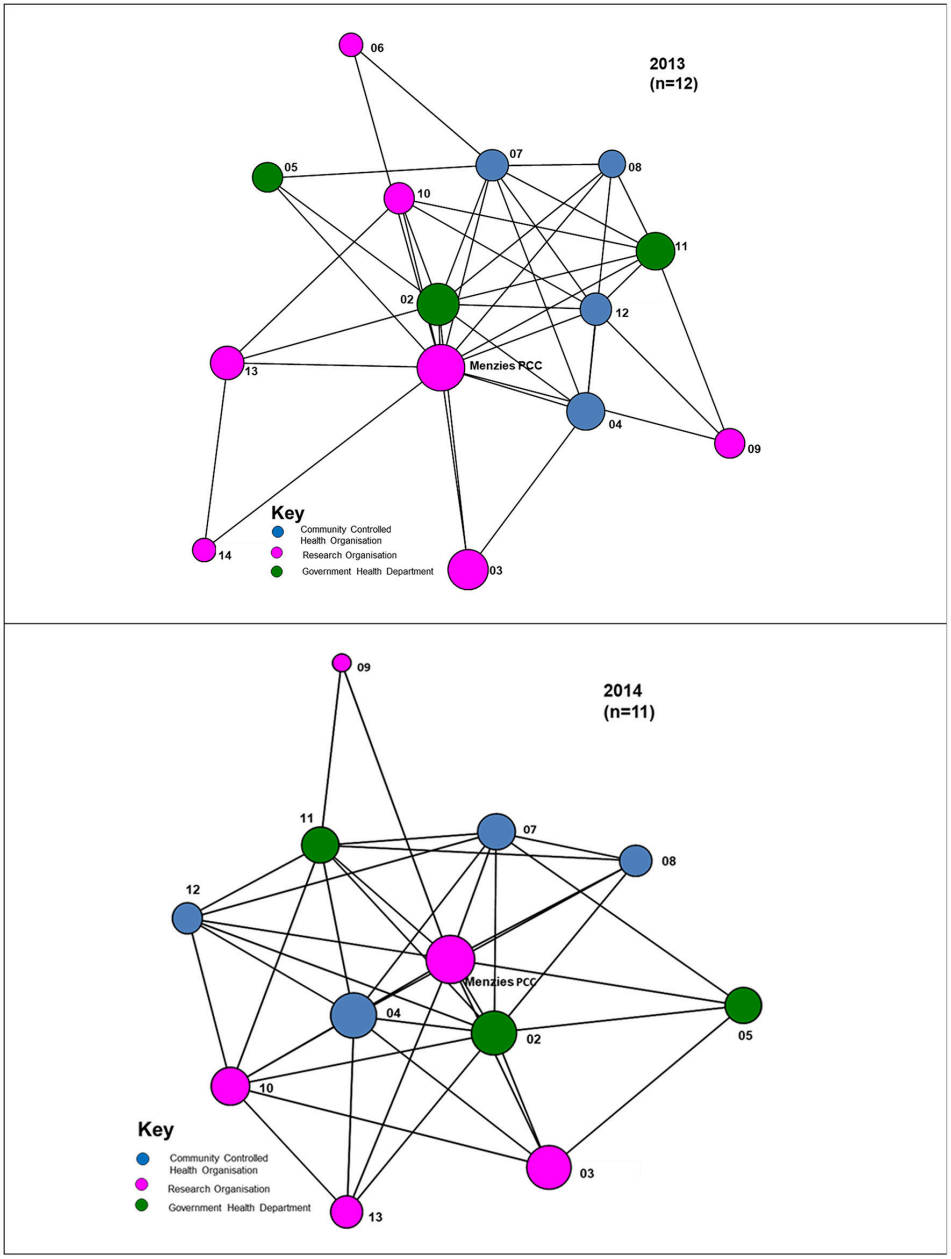
Overall “value” of members as perceived by other organisations with which they have connections in the Partnership, 2013, 2014.

Figure 6 shows the overall “value” of members of the Partnership, as perceived by other organisations with which they have connections in the Partnership. In 2013, members reported that the PCC was perceived as having the highest value of partner organisations in the Partnership. This is consistent with the central, organising and coordinating role of the Project Coordination Centre. However, members also valued other organisations highly, including an academic organisation and a jurisdictional health department. In 2014, the Centre was still perceived as having the highest value amongst partner organisations, and a community-controlled health organisation and a jurisdictional health department were additional organisations rated highly.

**Figure 7:**
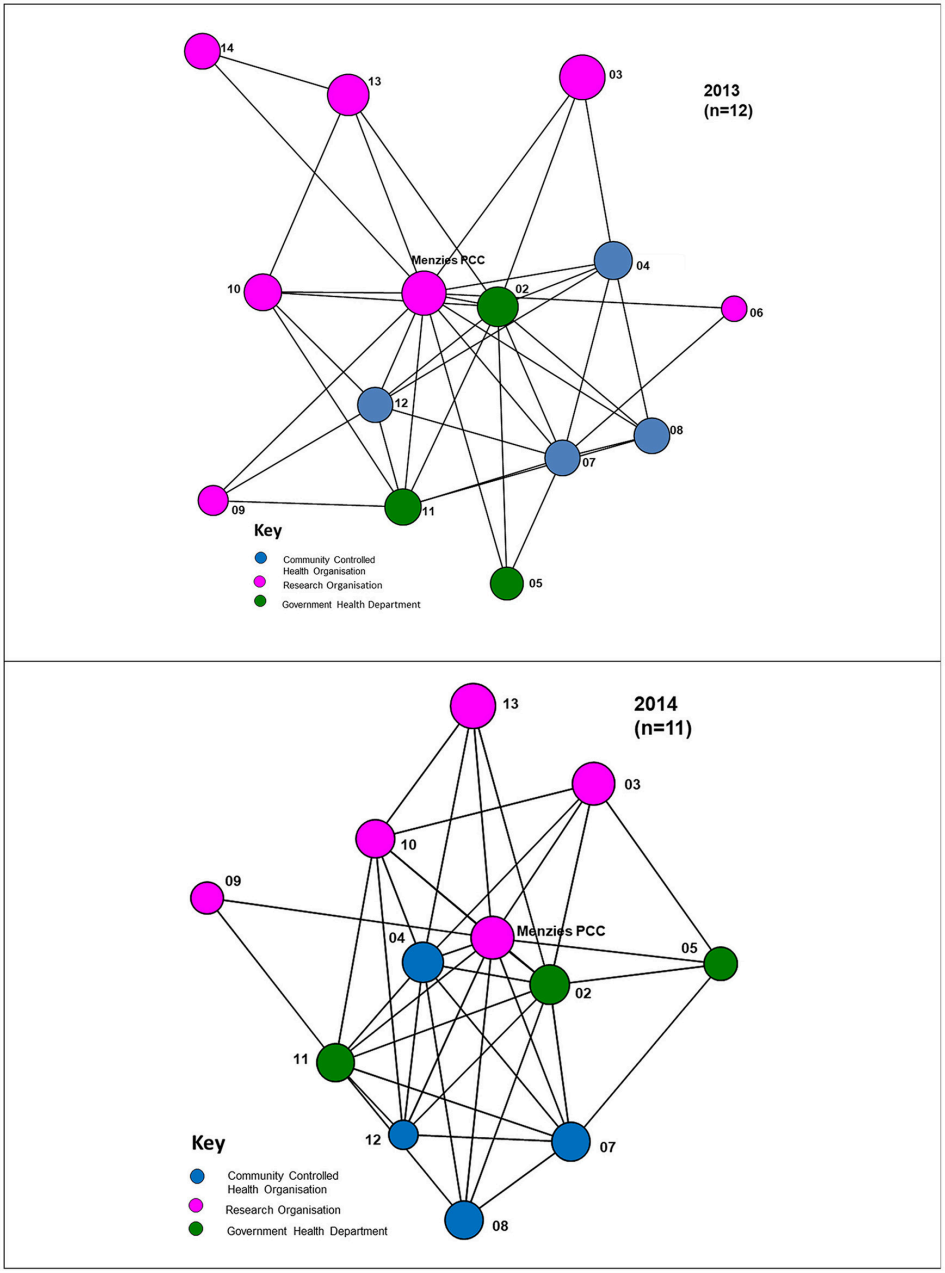
Overall “trust” of members as perceived by others with whom they have connections in the Partnership, 2013, 2014.

Trust functions as a facilitator for cooperation (34), and existing theories stress the role of social networks in shaping trust relations (35). As measured through the Partner Tool, the overall trust value for the Partnership was 70.1% in 2013, and 78.8% in 2014, indicating an increase in the overall level of trust from 2013 to 2014 (Figure 7). This shows a high level of trust across the network as a whole. A high level of trust is a key characteristic of a properly-functioning partnership allowing for effective information exchange (36). The size of the node for each partner organisation reflects the level of trust reported in that organisation by the other partners (the larger the node, the higher the level of trust).

## Discussion

### Key study findings in relation to study aims

This study aimed to apply social network methods to assess collaboration and functioning of the Partnership in 2013 and 2014. Partners perceived that the Partnership was successful in achieving its goals. The major element for success in its goals was its focus on the development of a shared database of de-identified clinical audit data from Indigenous health centres for quality improvement. Significant resource contributions that partners made to the Partnership included staffing, expertise, data collection and analysis, and leadership, elements that reflect their active involvement in the Partnership. Both leadership and advocacy were noted as important resource contributions from partners, and were regarded as a shared responsibility horizontally across partners. The Partnership was established to leverage the range of successful research collaborations that had flourished through the ABCD program of work from its commencement in 2002. In its turn, the Partnership provided a foundation of collaborative efforts across a range of partners to leverage the work of the CRE in Integrated Quality Improvement in Indigenous Primary Health Care which commenced in 2015 as an Innovation Platform.

With respect to identifying the major contributors to the success of the Partnership, partners highlighted the importance of exchanging information and knowledge, of partners having the opportunity to guide research and development efforts, and the informal relationships created through the Partnership. For achieving Partnership outputs, partners perceived as important the face-to-face biannual meetings, Partnership reports and publications and jurisdictional steering committee meetings. Due to resource constraints in the final operational year of the Partnership, it was possible to hold only one meeting in October 2014. The 2014 survey was conducted in mid-2014, and the lack of a face-to-face meeting in the first half of 2014 may have negatively influenced some of the study findings. Both formal and informal networking opportunities were also seen as a collaboration benefit.

It is important to recognise the context of a partnership, and this Partnership's context related to CQI in Indigenous primary health care. Partner organisations included ACCHOs, Indigenous peak bodies, academic research organisations and government departments in nearly all Australian jurisdictions. However, across the Partners individual participants in the Partnership included CQI facilitators and coordinators, PHC clinicians and health professionals, policy makers and academic researchers. The Partnership demonstrated strong engagement of Indigenous people in the PCC, in its regular developmental meetings, in the co-design and co-leadership of research, and in Partnership projects.

The network analysis found that network density increased (43% to 59%) from 2013 to 2014, demonstrating an increase over time in the connectivity and communication between partner organisations. Degree centralisation decreased from 67% to 49%, reflecting a shift during the life-cycle of the Partnership toward other partner organisations taking on more responsibilities for coordinating project work with partners, in addition to the core coordinating role of the Partnership Coordination Centre.

### How do findings relate to other research?

The finding of the importance to the research partnership of collecting and sharing data is supported by other research. Aveling et al. identified the importance to clinical practice networks of collecting and using data wisely (37). Regular feedback on performance can motivate sustained efforts by providing a sense of progress or keeping participants “on task”—particularly in between face-to-face gatherings (38) and when provided by peers (39). The finding relating to the importance of the regular face-to-face biannual Partnership meetings is consistent with findings in the literature supporting the use of face-to-face meetings for partnerships, as such elements of participation add value to the group (40). We noted the importance of recognizing the context of the Partnership working to enhance CQI in Indigenous PHC. Various authors have also identified the importance of contextual issues that are likely to be significant for clinical and health networks, including the particular health issue, the disciplines and specialisms involved (39, 41) and the local context of participants (42, 43).

While network density increased from 0.43 in 2013 and 0.59 in 2014, it should be noted that density depends on the size of the network, with larger networks, all other things being equal, having lower densities than small networks (44, p. 74). Density is also related to the type of network. Important contexts include geography, types of collaborative leadership, funding, governance structures, and missions.

The Partnership's degree centralization scores of 0.67 in 2013 and 0.49 in 2014, show a fairly high level of centralisation compared with findings for similar collaborations which range from 0.00 to 0.30 (22). The decrease in degree centralization from 2013 to 2014 reflects the shift toward other partners taking on additional coordination responsibilities in addition to the PCC. It is important for partnerships to nurture the growth of productive collaboration between their partners. Our finding is supported by evidence from a meta-analysis of network data collated from the Partner tool on public health partnerships. This analysis identified the importance of using systems-focused network leadership strategies to continue nurturing productive partnerships and to facilitate partnerships (45).

The important role of face-to-face meetings in the Partnership was identified in findings of research on quality improvement collaboratives which found that most involve face-to-face contact, sometimes supported by online, email or telephone support between meetings (9, p. 20).

### Possible study limitations

In interpreting study results, several possible study limitations need to be considered. There was a difference in Partnership membership and in the number of respondents in the two survey years. For the 2013 survey, 12 out of 14 partners responded to the survey, compared with 11 out of the 12 active partners in 2014. Even though there were few non-respondents, as the numbers are small in this study it is possible that the responses of non-respondents may have changed the response ratings on questions.

One of the limitations of the Partner tool software is that it is only possible to include one survey respondent from each partner organisation. As it is not possible to include people in the survey who are involved in the Partnership at other levels or areas of the organisation, this means that the full extent of a partner's involvement in the collaboration, and network connections, may not necessarily be reported by the one respondent for each partner. The diversity of views among other stakeholders in each organisation may not necessarily be reflected, and responses may not necessarily be representative of the views of the range of key players in the organisation. To capture wider perspectives on the functioning of similar research partnerships, future network research on research partnerships should ensure that all individuals participating in the partnership are included in the survey list. Network studies of partnerships can be specifically designed, along with a tailored network survey and tailored network analysis, instead of using an existing packaged SNA tool such as the Partner tool. However, such design and application of SNA methodologies requires SNA expertise, whereas the readily available Partner tool can offer ease of application, and potential utility to users where such expertise may not be readily available.

It is important that this network analysis of the formal signatories to the Partnership is located within the broader network of the Partnership which has been vital to its effectiveness. A separate assessment of the impact of the Partnership, which has assisted us in interpreting the findings from the current study, identified that between 2010 and 2014, 175 PHC centres provided the Partnership with de-identified clinical audit data derived from the use of CQI tools and processes (31). In addition, the Partnership brought together almost 60 stakeholder organisations—ACCHOs, government managed health centres, research institutions, government health departments, key regional support organisations such as Aboriginal community-controlled peak bodies and Medicare Locals (regional PHC structures)—from across jurisdictions and all levels of the health system to support and guide research on priority PHC systems issues. The Partnership also provided a vital foundation to securing funding for a new Centre of Research Excellence for an Innovation Platform for Integrated Quality Improvement in Indigenous Primary Health Care (National Health and Medical Research Council No. 1078927) (46).

### Implications for policy and practice and further research

Study findings on the success of the Partnership in achieving its goals, as perceived by its partners, are encouraging from the perspective of identifying successful approaches for research partnerships to improve CQI in Indigenous PHC. This study shows that the traditional social survey approach can be combined with the use of SNA tools to examine how a partnership is functioning. It was useful to the Partnership to investigate its functioning at mid-term so that findings could be fed back to the Partnership to develop forward strategies. The second survey in the final year of the Partnership lifecycle allowed for comparison of the Partnership at two timepoints, and allowed for the collection of partner perceptions addressing the full duration of the Partnership.

The Partnership extended across the majority of Australian jurisdictions and involved a wide range of partners involved in Indigenous PHC. The structure of the Partnership included regional research hubs based in jurisdictions with representation from the range of partners to ensure that research reflected priority local issues. The Partnership provides a model for other research partnerships aiming to have an impact on applying research to improve practice in PHC, and more broadly in other collaborative health research.

## Conclusion

Three key factors have been identified as important to effective management of networks: effective collaboration must be secured; the right assembly of resources must be achieved; and shared sense-making must be created amongst the people involved (47). For the Partnership, firstly, the network measures of collaboration show that at both time points the Partnership was a highly connected network. Network connectivity and communication, as measured by network density, increased from 43 per cent to 59 per cent, reflecting effective collaboration. Secondly, partners contributed a range of resources to the partnership and these were coordinated through the PCC. Thirdly, survey feedback from partners indicated that there was a high level of agreement overall on achievement of the key goals of the Partnership.

Partners reported that key attributes of the Partnership contributing to its success included: the opportunity to guide research and development, exchanging information / knowledge and the informal relationships created. These are key learnings from the Partnership that can be carried forward in future research collaborations in quality improvement in Indigenous PHC, as well as more broadly in other collaborative applications.

The work of the Partnership is continuing through the Centre of Research Excellence. Over 5 years it will leverage the efforts of researchers, service providers and policy-makers to address priority areas for development of integrated quality improvement in Indigenous PHC. It expands upon the partnership model established through the ABCD Partnership, and involves a wider range of participants and organisations than the initial partners who were participating organisations in the Partnership reported on in this paper.

## Author contributions

FC was primarily responsible for study conduct and coordination and for the initial draft of the manuscript. FC and VM had primary responsibility for the development and conduct of the respondent survey, and for the synthesis and analysis of material from the three study phases. All authors contributed to the ideas in this paper: they provided input into the study conceptualisation and the development of the evaluation framework. All authors provided editorial contributions and read and approved the final manuscript.

### Conflict of interest statement

The authors declare that the research was conducted in the absence of any commercial or financial relationships that could be construed as a potential conflict of interest.

## Abbreviations

ABCD: Audit and Best Practice for Chronic Disease program
ACCHO: Aboriginal Community Controlled Health Organisation
CQI: continuous quality improvement
CRE: Centre of Research Excellence
IQI: integrated quality improvement
PCC: Project Coordination Centre
PHC: primary health care
QI: quality improvement
SNA: social network analysis
US: United States.
