# Supplementary material for: Assessing Collaboration in a National Research Partnership in Quality Improvement in Indigenous Primary Health Care: A Network Approach
Source: Front Public Health. 2018 Jun 25;6:182. doi: 10.3389/fpubh.2018.00182 (PMC6026655; doi:10.3389/fpubh.2018.00182)
Supplement: Supplementary file 1 [file Table_1.DOCX]

**Appendix A**

Table 1: Collaborative survey questions

| **Summary of questions in Survey, 2013 and 2014** |
| --- |
| Demographic / organisational questions: |
| - Job title? - Current position within organisation/department? - Length of time as a member of the Partnership? - Resources contributed to the Partnership? - Most important contribution or potential contribution to Partnership? |
| Perceptions of Partnership Project mission and success |
| - Most important outcomes of the Partnership? - At this stage of the project, how successful has the Partnership been at progressing to its objective overall? *Not successful, Somewhat Successful, Successful, Very Successful* |
| Outcome questions |
| - Partnership outcomes? - Most important outcome? - At this stage of the project, how successfully has the Partnership progressed? - Factors contributing to successful outcomes? |
| Relational Questions |
| *Please list all organisations with whom you have a relationship with to meet the goals of the Partnership.*  For each organisation chosen, the following relational questions were asked:   1. Frequency of interaction (*None, once a year*) 2. Types of interactions? 3. Level/quality of interaction (*Networking, cooperative, coordinated, integrated activities*) 4. Extent of value as:    1. power/influence;    2. level of involvement;    3. resource contribution. 5. Extent of trust as:    1. reliable;    2. in support of mission;    3. open to discussion. |
| Additional Questions 2014 |
| - Perceptions of the benefit of Partnership processes and outputs - Perceived benefits of collaboration in Partnership - Partners perceptions of achievement of goals - Perceived level of success: subsidiary goals |
